# The Laboratory Text-Book of Public Health

**Published:** 1898-06

**Authors:** 


**The Laboratory Text-Book of Public Health.** By William R. Smith, M.D. Pp. xx., 322. London: Henry Renshaw. 1896.

This book is intended to serve as laboratory companion to candidates for the diploma in public health and aims at giving concise but sufficient descriptions of such processes as are required to be performed in the practical part of this examination. This intention is on the whole admirably carried out. The descriptions both of apparatus and of methods are very clear and intelligible, and the selection of the latter is well adapted to the requirements of the case.

The student is perhaps left a little in the dark as to the meaning of the analytical data he may have obtained from a given sample of water; but possibly the interpretation of results is not considered to be within the scope of the work. Water analysts, too, will hardly endorse the twice repeated statement that nitrates cannot be qualitatively detected in the presence of nitrites; and the process given for the detection of phosphoric acid is faulty in several respects: but with these exceptions the section on water analysis, one of the most important to the candidate, is well worked out.

The succeeding chapters on gas analysis, on the examination of foods, and on bacteriology are very judiciously written, all that is necessary, except perhaps Widal's serum test for typhoid, being presented in condensed but clearly expressed language.

Photo-micrographs of the commoner starches supplement the text, and will prove of great assistance.

It will be understood, of course, that for the written examination much additional reading will be necessary, but for the practical work it would be difficult to find a more appropriate guide.

